# Antifungal Activity of the Frog Skin Peptide Temporin G and Its Effect on *Candida albicans* Virulence Factors

**DOI:** 10.3390/ijms23116345

**Published:** 2022-06-06

**Authors:** Felicia Diodata D’Auria, Bruno Casciaro, Marta De Angelis, Maria Elena Marcocci, Anna Teresa Palamara, Lucia Nencioni, Maria Luisa Mangoni

**Affiliations:** 1 Department of Public Health and Infectious Diseases, Laboratory Affiliated to Istituto Pasteur Italia Fondazione Cenci Bolognetti, Sapienza University of Rome, 00185 Rome, Italy; felicia.dauria@uniroma1.it (F.D.D.); marta.deangelis@uniroma1.it (M.D.A.); mariaelena.marcocci@uniroma1.it (M.E.M.); annateresa.palamara@uniroma1.it (A.T.P.); 2 Department of Biochemical Sciences, Laboratory Affiliated to Istituto Pasteur Italia Fondazione Cenci Bolognetti, Sapienza University of Rome, 00185 Rome, Italy; bruno.casciaro@uniroma1.it; 3 Department of Infectious Diseases, Istituto Superiore di Sanità, 00161 Rome, Italy

**Keywords:** antimicrobial peptides, Temporin G, antifungal activity, *Candida albicans*, dermatophytes, *Aspergillus*, yeast-mycelial conversion, biofilm, mechanisms

## Abstract

The increasing resistance to conventional antifungal drugs is a widespread concern, and a search for new compounds, active against different species of fungi, is demanded. Antimicrobial peptides (AMPs) hold promises in this context. Here we investigated the activity of the frog skin AMP Temporin G (TG) against a panel of fungal strains, by following the Clinical and Laboratory Standards Institute protocols. TG resulted to be active against (i) *Candida* species and *Cryptococcus neoformans,* with MIC_50_ between 4 µM and 64 µM after 24 h of incubation; (ii) dermatophytes with MIC_80_ ranging from 4 to 32 µM, and (iii) *Aspergillus* strains with MIC_80_ of 128 µM. In addition, our tests revealed that TG reduced the metabolic activity of *Candida albicans* cells, with moderate membrane perturbation, as proven by XTT and Sytox Green assays, respectively. Furthermore, TG was found to be effective against some *C. albicans* virulence factors; indeed, at 64 µM it was able to inhibit ~90% of yeast–mycelial switching, strongly prevented biofilm formation, and led to a 50% reduction of metabolic activity in mature biofilm cells, and ~30–35% eradication of mature biofilm biomass. Even though further studies are needed to deepen our knowledge of the mechanisms of TG antifungal activity, our results suggest this AMP as an attractive lead compound for treatment of fungal diseases.

## 1. Introduction

The past two decades have seen a dramatic increase in fungal infections, causing an emergency, particularly in hospitalized patients and immunocompromised individuals [1,2]. The most prominent pathogens responsible for superficial and mucosal infections with frequent recurrence are *Candida* strains [3]. Furthermore, invasive fungal infections, especially in a hospital setting, are progressive and lethal if not diagnosed and specifically treated [4]. *Aspergillus*, *Cryptococcus*, *Candida*, and *Pneumocystis* are the main types responsible for severe infections [5]. On the other hand, the increase of a variety of fungal strains resistant to conventional drugs has become a significant problem [6], and some medical treatments struggle to treat and fully eradicate fungal biofilm-related infections [7,8]. Therefore, there is an urgent demand for the identification of new agents with different targets or mechanisms of action. Antimicrobial peptides (AMPs) are a class of molecules widely distributed in nature [9,10]. They are present in both prokaryotic and eukaryotic organisms and are critical components of the innate immunity, with the function of protecting the host against a wide range of microorganisms, including bacteria, fungi, protozoa, and viruses [9,11,12]. Many works have demonstrated the effectiveness of AMPs against a broad spectrum of microbial pathogens [13,14,15]. Among the AMPs are found temporins, short peptides initially isolated from the skin secretion of the European red frog *Rana temporaria* [16,17,18,19,20,21] and subsequently also identified in other ranid frogs of both North American and Eurasian origin [22]. Temporins A, B, and L are among the most studied isoforms and their activity has been well demonstrated against both Gram-positive and Gram-negative bacteria, viruses, and *Candida* [20,21,23,24,25]. More recently, our group has focused attention on the isoform temporin G (TG), which has been found to inhibit replication of two respiratory viruses, influenza and parainfluenza, by interfering with the viral entry into the cells, as well as to kill *Staphylococcus aureus* biofilm [26,27].

Based on the wide spectrum of antimicrobial activity of TG and the unexplored efficacy of this peptide and temporins, in general, especially against filamentous fungal species, we evaluated the activity of TG against different pathogenic fungi, such as dermatophytes and *Aspergillus* spp.; as well as against different *Candida* species, for comparison. Furthermore, we investigated the effect of TG on some virulence factors of *C. albicans*, and performed preliminary tests to understand the molecular mechanism of TG. *C. albicans* is an opportunistic pathogen involved in many human infections [1]. Among the virulence factors that contribute to fungal pathogenesis, the most significant feature is the morphological yeast-to-hyphal transition. Indeed, it facilitates a rapid response to different environments, as well as during host infection [28]. Moreover, the ability of C. *albicans* to change morphology is also crucial for biofilm formation [29,30]; a complex structure, in which a community of microbial cells embedded in an extracellular matrix is linked to host tissue or abiotic surfaces. Remarkably, biofilm formation strengthens pathogenesis, causing high morbidity and mortality and makes *Candida* more tolerant to antimicrobial therapy [31].

## 2. Results

### 2.1. TG Exerts Activity on Different Fungal Strains

The results of TG activity against a panel of fungal strains are reported in Table 1 and Table 2. Basically, the growth inhibitory activity was studied according to the standard protocol established by the Clinical and Laboratory Standards Institute [32,33].

Although intraspecies variability was observed, we found a good activity against *C. albicans* and *C. tropicalis,* with MIC_50_ ranging from 4 to 16 µM after 24 h of incubation, and between 8 and 32 µM after 48 h. Total visual growth inhibition (MIC_100_) was achieved at peptide concentrations of 16 and 32 µM after 24 and 48 h of incubation, respectively. Note that these concentrations were lower than the concentration causing 50% cytotoxicity (73 µM) against human mammalian cells [26].

Conversely, TG was less effective when tested against other non-*albicans Candida* species; in fact, MIC_50_ ranged from 16 to 64 µM after 24 h, and from 32 to 128 µM after 48 h (Table 1). In comparison, the MIC_100_ was obtained at concentrations ranging from 32 to 128 µM, after either 24 h or 48 h of incubation (Table 1). Notably, the MIC of TG against some strains with resistance to reference antifungal drugs (such as amphotericin B) was similar to that obtained for the sensitive microbes.

TG was also active against *Cryptococcus neoformans,* with MIC_50_ of 16 µM; MIC_100_ was obtained at 32 µM, and no variation was observed at different incubation times (Table 1).

We also evaluated the effect of TG on dermatophyte strains (Table 2). The peptide showed a good activity against *Trichophyton* spp. and *Microsporum* spp. between 4 and 32 µM. The mean MIC_80_ was 19 ± 20.3 µM for *Trichopyton* spp. and 27.7 ± 13.9 µM for *Microsporum* spp. The MIC_100_ was recorded between 16 and 128 µM peptide concentrations. The mean MIC_80_ was 42 ± 28.7 µM for *Trichopyton* spp. and 101.3 ± 32.2 µM for *Microsporum* spp. 

Moderate activity was observed against three *Aspergillus* species, with a mean MIC of 112 ± 28.3 µM and maximal growth inhibition at a mean MIC of 124.9 ± 49.6 µM. The three selected strains were resistant to amphotericin B, having a mean MIC of 12.7 ± 9.1 µM (Table 2).

### 2.2. Fungal Cell Viability Decreases at Increasing Concentrations of TG

The viability of *Candida* cells was evaluated by methylene blue assay in a nutrient-free medium, to determine if TG could affect cell viability after a short period of contact.

**Figure 1:**
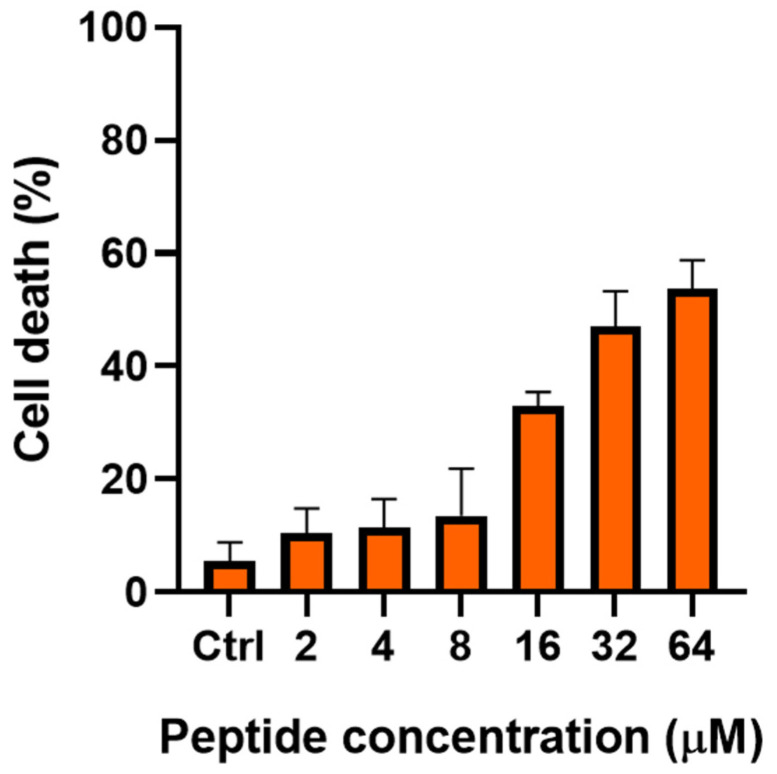
Effect of TG on the viability of *C. albicans* ATCC10231. Cell viability was estimated with methylene blue dye. The values are presented as the percentage (%) of dead cells with respect to untreated control cells (0% cell death) after 6 h of treatment. Values are mean ± SD of three independent measurements, each performed in duplicate (n = 6).

This dye penetrates all cells, but viable cells enzymatically reduce the dye to a colorless product and become unstained, whereas dead cells are stained blue. The extent of cell death was determined by manually counting the number of colorless vs. blue cells in the sample. As reported in Figure 1, the percentage (%) of blue-stained *C. albicans* cells directly correlated with the peptide concentration. In particular, the percentage of blue cells was ~30% after 6 h treatment with TG at 16 µM; ~45% after treatment with TG at 32 µM; and ~54% when TG concentration was equal to 64 µM.

### 2.3. TG Induces Membrane Perturbation

**Figure 2:**
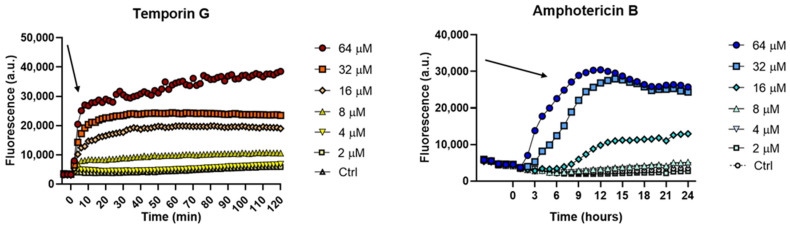
Kinetics of membrane permeabilization of *C. albicans* ATCC10231 strain induced by the addition of TG and amphotericin B (t = 0) at different concentrations. Arrows indicate the maximum perturbation effect that occurred in the first minutes or 6 h after peptide/antibiotic addition, respectively. Samples were incubated with 1 μM Sytox Green in 0.01 M sodium phosphate buffer (NaPB), and changes in fluorescence were monitored. Controls were microbial cells with vehicle. Values correspond to one representative experiment of three.

The kinetics of cytoplasmic membrane perturbation was evaluated using a Sytox Green assay. This probe is impermeable to intact membranes, and it increases its fluorescence when bound to nucleic acids. As reported in Figure 2, the addition of TG (T = 0) caused a dose dependent increase of fluorescence intensity within the first minutes, at concentrations of 16, 32, and 64 µM, indicating a fast destabilization of the membrane, in line with previously published reports for various AMPs [27,34,35]. This would allow the intracellular influx of the dye, with a consequent binding to nucleic acids and increase of fluorescence. A slight perturbation effect was visible at 8 µM, while the low concentrations of 2 and 4 µM of TG were inactive. Amphotericin B, which was used as reference drug, showed a similar trend, but with much slower kinetics. In fact, the maximum perturbation effect occurred after 6 h (indicated by the arrow in Figure 2) from the addition of the compound.

**Figure 3:**
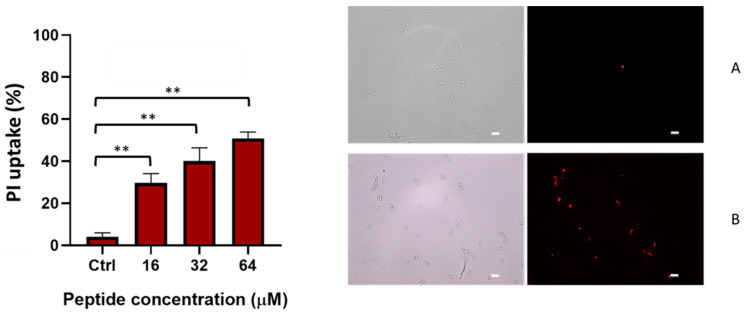
Effect of TG on the membrane permeability of *C. albicans* ATCC10231. Membrane permeability of *Candida* cells after 6 h treatment with TG was evaluated using PI uptake. The percentage of cells stained in red versus the untreated control (Ctrl) was evaluated. Three replicates were performed, and values were reported as mean ± SD. Images are of untreated Ctrl (**A**) and 64 µM TG-treated cells (**B**). Differences between TG-treated cells and Ctrl are significant (** *p* < 0.01). Scale bar, 10 μm.

The data of Sytox Green were confirmed using a propidium iodide (PI) assay performed for a longer period (6 h). PI is a membrane-impermeant nucleic acid stain that enters cells with compromised membranes. It binds DNA and emits red fluorescence upon excitation. As shown in Figure 3, a concentration-dependent PI uptake was found in *C. albicans* cells treated with TG.

Indeed, at 16 µM, the percentage of PI-positive treated cells was ~30% and increased up to 50% at 64 µM, compared to the 4% observed in the untreated control samples. These results support the notion that the membrane integrity of *Candida* cells is injured by TG in a dose-dependent manner.

### 2.4. TG Does Not Directly Bind Ergosterol

**Figure 4:**
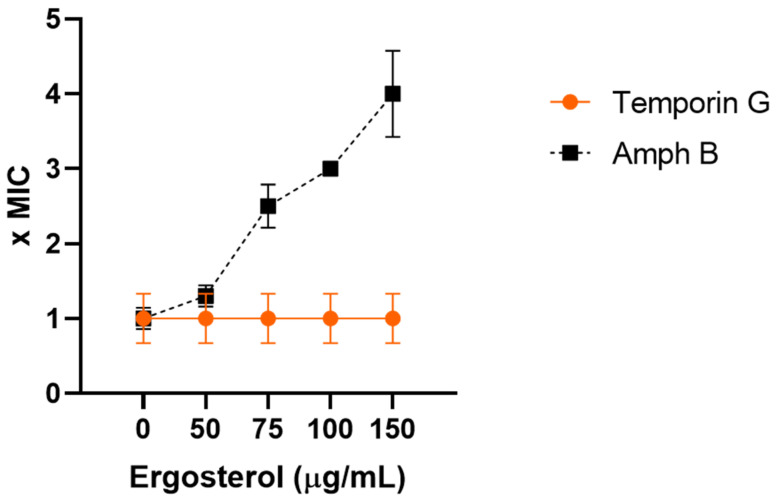
Effect of exogenous ergosterol on the anticandidal activity of TG and amphotericin B. MIC values were determined according to CLSI, in the absence and presence of different concentrations (50–150 µg/mL) of exogenous ergosterol. The MIC of TG and amphotericin B against *C. albicans* ATCC10231 was evaluated after 48 h of incubation at 37 °C.

Ergosterol is a well-known component of the fungal cell membrane, and it is an important target of many antifungal drugs [36]. We, therefore, investigated the possible binding of TG to ergosterol, by evaluating the MIC of the peptide in the presence of exogenous ergosterol (see Section 4) [37,38]. As shown in Figure 4, the MIC of TG remained unchanged, while the MIC of amphotericin B, a well-known antifungal drug that interacts with the cell membrane ergosterol [36], was enhanced in parallel with increasing concentrations of exogenous ergosterol. These data suggest that TG does not bind directly to the ergosterol of the fungal cell membrane.

### 2.5. TG Does Not Affect Fungal Cell Wall Synthesis

Finally, we investigated the possible involvement of TG on the cell wall synthesis through a sorbitol assay. Sorbitol is an osmotic protectant used in the stabilization of protoplasts [39], and when it is added to medium, the MIC of inhibitors of cell wall component biosynthesis rises remarkably [40]. In our model, when sorbitol was added to the medium, the MIC of TG was not increased compared to the MIC obtained without sorbitol (Table 3), suggesting that TG does not interfere with the fungal cell wall synthesis. Conversely, caspofungin, which is a well-known inhibitor of β(1,3) glucan synthesis, significantly increased the MIC in the presence of sorbitol (Table 3).

### 2.6. TG Is Active against Some Candida Virulence Factors

#### 2.6.1. Inhibition of Yeast-Mycelial Conversion

**Figure 5:**
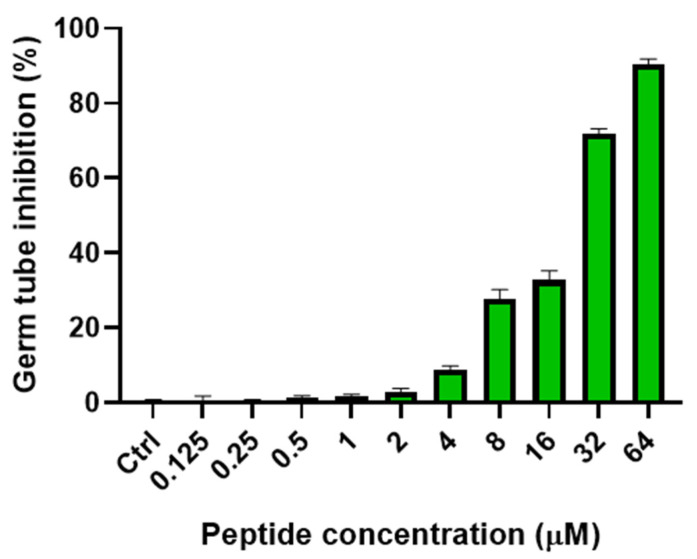
Effect of different concentrations of TG on *C. albicans* ATCC10231 yeast–mycelial switching. The effect of TG on yeast–mycelial switching was evaluated by visually counting germ tube formation, the first step of mycelial formation. After 3 h of incubation at 37 °C in GlcNAc solution under shaking, the percentage of germ tubes with respect to the total cells was evaluated microscopically, by counting cells from each sample. The assay was performed in duplicate, and the values are expressed as the mean of three independent experiments ± SD.

In response to different environmental signals, *C. albicans* can grow as yeast, pseudohyphae, or true hyphae [41]. The transition from yeast to mycelium is an important virulence factor [42]. In fact, some studies have demonstrated that the mycelial form has a strong adhesion ability and can easily invade host cells causing inflammation [43,44]. Owing to its importance in pathogenicity, yeast–mycelial switching in *C. albicans* is of great interest for the identification of new antifungal drugs. Thus, the effect of TG on the yeast–mycelial transition was studied in *C. albicans* ATCC10231, and the results are reported in Figure 5. We analyzed the transition by observing germ tube forming cells in GlcNAc solution (see Section 4). The effect of TG was dose-dependent, and a prominent activity was obtained at 16 µM, with ~31% inhibition of germ tube formation. At 32 µM, TG inhibited 72% of germ tube formation, while a remarkable activity was displayed at 64 µM, with ~90% of germ tube inhibition (Figure 5).

#### 2.6.2. Antibiofilm Activity of TG

We tested the activity of TG on *C. albicans* ATCC10231 biofilm formation and mature biofilm. Biofilm biomass and metabolic activities were evaluated using crystal violet (CV) and XTT assays, respectively.

**Figure 6:**
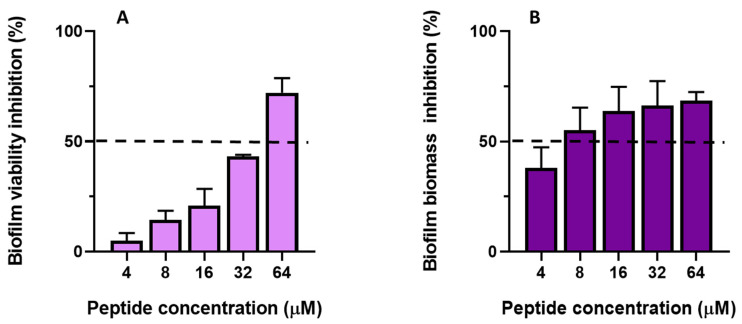
Effect of TG on the inhibition of *C. albicans* ATCC10231 biofilm formation. The effect of TG on the formation of *C. albicans* biofilm was evaluated using the XTT reduction assay (**A**) and CV assay (**B**). The assay was performed in duplicate, and the values are the mean of three independent experiments ± SD for XTT assay. CV assay was performed in triplicate and the values are the mean of two independent experiments ± SD. The values are expressed as percentage (%) of inhibition of metabolic activity (**A**) and percentage of biofilm inhibition (**B**) normalized to that of untreated control cells (0% inhibition).

When biofilm formation was evaluated, data revealed that TG impaired both the metabolic activity and biofilm biomass in a dose-dependent manner (Figure 6A,B).

Indeed, the lowest concentration of peptide able to cause a 50% reduction of metabolic activity (defined as BIC_50_, dotted line in Figure 6A) compared to untreated samples was between 32 and 64 μM. At lower concentrations (i.e., 4, 8, and 16 μM), despite a reduction of metabolically-active cells in the range of ~5–21%, TG was able to strongly inhibit biofilm biomass (50% reduction at 8 μM, dotted line in Figure 6B). This suggests that during biofilm formation TG impairs not only germ tube formation (Figure 5) but also the production of the extracellular matrix.

**Figure 7:**
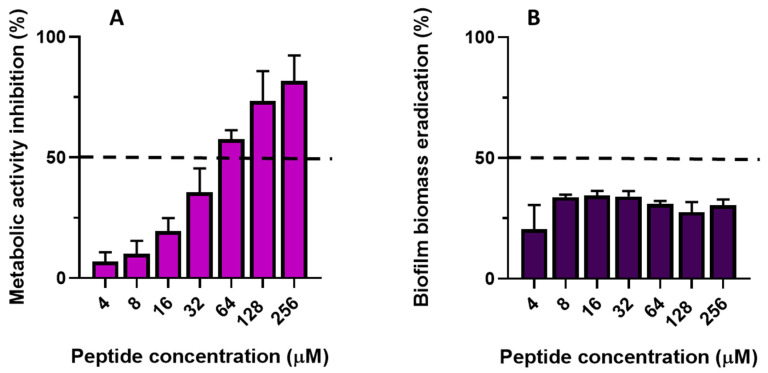
Effect of TG on the eradication of *C. albicans* ATCC10231 mature biofilm. The effect of TG on mature (24 h) biofilm of *C. albicans* was evaluated using an XTT metabolic reduction assay (**A**) and CV assay (**B**). The assay was performed in duplicate, and the values are the mean of three independent experiments ± SD for XTT assay. CV assay was performed in triplicate and the values are the mean of two independent experiments ± SD. The values are expressed as percentage (%) of inhibition of metabolic activity (**A**) and percentage of mature biofilm inhibition (**B**), normalized to that of untreated control cells (0% inhibition).

The effect of TG on 24 h mature biofilm is reported in Figure 7A,B. TG reduced the number of viable cells; indeed, 74% and 81% inhibition of metabolic activity occurred at 128 μM and 256 μM, respectively (Figure 7A). The lowest concentration of peptide able to cause 50% reduction of metabolic activity in biofilm cells (defined as BEC_50_, dotted line in Figure 7A) was between 32 and 64 μM. In comparison, when biofilm biomass was evaluated, TG was found to cause ~30–35% biomass eradication (Figure 7B), with no significant differences in the activities among concentrations.

## 3. Discussion

*Candida* spp., *Cryptococcus neoformans*, dermatophytes, and *Aspergillus* spp. are microorganisms responsible for most superficial and systemic infections, particularly in patients with a compromised immune system [45]. *C. albicans* is a commensal microbe of the normal microbiota of healthy humans [46], but is responsible for a high mortality rate among all nosocomial bloodstream infections [47,48,49]. Indeed, *Candida* spp. express several virulence factors that enable them to invade host tissues and cause various infections, especially when the host immune system is hampered [50,51]. One of these virulence factors is provided by the formation of biofilm, a complex matrix-enclosed community of cells [52]. In the early stage of *Candida* biofilm formation, single cells adhere to a biotic or abiotic surface; then a complex network of hyphae, pseudohyphae, and yeast cells is produced, to finally form a mature biofilm surrounded by an extracellular matrix [53]. Notably, biofilm favors dissemination of fungal cells and protection of cells from the host immune clearance [54], making them less susceptible to many antifungal drugs [55]. Most of the fungal infections in hospitalized patients are strictly connected to biofilm formation on medical devices [56] and directly correlate with increased mortality [31,57,58]. Therefore, biofilms are an important threat for human disease, especially for their resistance to antifungal drugs. The available antifungals are not efficient in eradicating biofilm-related infections [59,60,61], and the emergence of multi-resistant fungal strains [62,63,64] has become a serious health concern in this century [65,66]. In recent years, the search for new therapeutic approaches with different targets or mechanisms of action has been constantly evolving [64,67], and several authors have proposed AMPs, and temporins, as promising scaffolds for the development of new antifungal compounds [13,14,16,23,24,25,68]. Our group and others have described the antifungal activity of temporins L and B [69,70]. A series of cyclic analogs of temporin L was tested against a panel of *Candida* species with MIC of 50 µM. Furthermore, in comparison, some isoforms of temporin L were able to provoke an almost complete inhibition of biofilm formation and/or eradication at 25 µM. The same authors also hypothesized a local membrane permeabilization, with a type of carpet model, as the plausible mechanism of fungicidal activity [70]. Recently, a significant antifungal activity of temporin SHa analog was also observed, against a panel of yeasts and molds of clinical interest, with a synergistic effect when combined with amphotericin B [71].

In this study, we demonstrated that TG has a wide range of antifungal activity against *Candida* species and *Cryptococcus neoformans*, at concentrations non-toxic for human cells [26,27]. Interestingly, TG also showed activity against dermatophytes at concentrations ranging from 4 to 32 µM, and against *Aspergillus* spp., although at higher concentrations (128 µM). To the best of our knowledge, this is the first demonstration of the effectiveness of temporins against a wide panel of fungal strains including *Candida*, *Cryptococcus*, and filamentous fungi belonging to dermatophytes and *Aspergillus* spp., while the reference amphotericin B showed a higher MIC against *Aspergillus* strains. These results are of great interest, due to the implication of these fungi in superficial or severe diseases, and due to the burden of antifungal resistance. In *Cryptococcus neoformans*, an opportunistic pathogen that causes meningitis with high mortality, particularly in HIV patients and other patients with defects in cell-mediated immunity, the current antifungal therapies have limitations because of their side effects and the emergence of resistance [72]. Similarly, in dermatophytes, filamentous fungi responsible for superficial infection (skin, nails, hair), antifungal treatment may be a failure because of the long-term therapy, frequent relapses, and the emergence of recalcitrant dermatophytosis [73,74]. *Aspergillus* spp. are ubiquitous saprophytic fungi and clinically relevant etiological agents of a wide spectrum of diseases, including allergic aspergillosis, lung infections, cutaneous aspergillosis, and severe invasive aspergillosis, mainly in immunocompromised patients. Treatment is carried out with azoles or amphotericin B, but has limited effectiveness, due to serious side effects and the frequent emergence of resistance [75]. Recently, two albumins-derived AMPs at 50 μg/mL demonstrated antidermatophytic activity against *Trichophyton* spp., causing morphological damage to cell walls and membranes of the hyphae [76]. The crotalicin peptide from a rattlesnake venom gland cathelicidin was found to significantly inhibit the growth of *Trichophyton rubrum* (MIC of 5 µM), but it was completely inactive against *Candida* [77].

We chose to investigate the molecular mechanism underlying the activity of TG against *C. albicans*, due to its implication in the epidemiology of severe fungal diseases, especially in nosocomial patients [78]. Our preliminary experiments suggested that TG does not interfere with *C. albicans* cell wall synthesis, but rather causes destabilization of the cell membrane structure, presumably by causing small local breakages, allowing the passage of small molecules, such as Sytox Green and PI (molecular weight of ~600), and not involving complexation with ergosterol, the main sterol component of the fungal membrane.

As previously described for other membrane-active temporins [18,79], TG may interact with the fungal cell membrane, causing a transient permeabilization without complete loss of membrane integrity. Furthermore, when tested at 32 µM, TG was able to inhibit the switch from the yeast to the hyphal form, with 70% inhibition compared to untreated cells; while at 64 µM, 90% inhibition was achieved. The ability of *C. albicans* to switch from the yeast to the hyphal growth forms (dimorphism) is a well-known virulence factor [37,80,81,82,83]. The hyphal form can easily penetrate the epithelial and endothelial host tissues [84] and escape from the immune system [85]; moreover, it is an important component of the structured mature biofilm [86,87,88]. As reported in the literature, the inhibition of the dimorphic transition in *C. albicans* can affect biofilm formation [89] and may be sufficient to treat candidiasis [90,91]. In our case, inhibition of *Candida* biofilm could be ascribed to the peptide ability to inhibit germ tube formation; thus, hampering adhesion of cells to surfaces with a reduction of network aggregation. This is in line with the reduced production of an extracellular matrix and number of viable biofilm cells shown in Figure 6.

We also observed that TG caused about a 50% reduction of metabolically-active cells in mature biofilm at a concentration between 32 µM and 64 µM; at the same concentration range, ~30% of biofilm biomass was eradicated. The activity did not depend on TG concentration. This agrees with the findings of Souza and collaborators, who recently reported the anti-*Candida* activity of two synthetic peptides (10 residues-long) PepGAT and PepKA, with a 30% reduction of mature biofilm at a comparable concentration [92]. Our data suggest that TG, while only partially eradicating preformed biofilm, can promote a significant reduction in the number of viable cells.

In conclusion, our results highlight the activity of TG against a wide panel of fungal strains, as well as its effectiveness in inhibiting the yeast to hyphal transition and in reducing biofilm formation in *C. albicans*. Even though further tests are needed to deepen our understanding of TG antifungal mechanisms, this peptide may be an attractive lead compound for the development of future alternative antifungal agents, likely to be used alone or in combination with conventional antifungal drugs, especially against yeast and filamentous fungal-induced diseases.

## 4. Materials and Methods

### 4.1. Fungal Strains

The evaluation of antifungal activity was carried out with strains coming from the American Type Culture Collection (ATCC, Manassas, VA, USA), from the German Collection of Microorganisms (DSMZ, Braunschweig, Germany), and the Pharmaceutical Microbiology Culture Collection (PMC, Sapienza University, Rome, Italy). The tests were performed on *Candida albicans* (ATCC10231, ATCC24433), *Candida krusei* (DSM6128, PMC0610, PMC0624), *Candida parapsilosis* (DSM11224, PMC0711), *Candida tropicalis* DSM11953, *Candida glabrata* PMC0805, *Cryptococcus neoformans* DSM11759, *Trichophyton mentagrophytes* (DSM4970, PMC6529, PMC6530), *Microsporum gypseum* (DSM3824, PMC7330), *Aspergillus brasiliensis* DSM1988, *Aspergillus niger* PMC7101, and *Aspergillus terreus* PMC7201.

### 4.2. TG and Antifungal Drugs

The activity of TG (Biomatik, Wilmington, DE, USA) was evaluated. The peptide was prepared in phosphate buffer saline pH 7.0 (PBS) at an initial concentration of 2 mM and successively diluted in medium at desired concentrations. Stock solutions of the antifungal drugs amphotericin B and caspofungin (Sigma-Aldrich, St. Louis, MO, USA) were prepared in 100% Dimethyl sulfoxide (DMSO) and further diluted in RPMI 1640 medium at the used concentrations. The solvent did not affect fungal growth.

### 4.3. Antifungal Susceptibility Testing

Antifungal susceptibility tests were performed according to CLSI guidelines for yeast and filamentous fungi, using the broth microdilution method with some modifications [32,33]. Candida strains and *Cryptococcus neoformans* were grown on Sabouraud Dextrose Agar (SDA) at 37 °C for 24 h and 48 h (*Cryptococcus*). The final concentration of the inoculum was 1 × 10^3^–5 × 10^3^ cells/mL. Dermatophytes were grown on Potato Dextrose Agar (Sigma Aldrich, St. Louis, MO, USA.) at 30 °C, until good conidial growth was present. The conidia suspension was prepared at a final concentration of 1 *×* 10^3^–3 × 10^3^ cells/mL [32]. The in vitro antifungal susceptibility was evaluated using TG at a concentration ranging from 0.5 to 256 µM and, as a reference drug, amphotericin B (drug concentration ranged from 0.125 to 64 µM). Caspofungin was used as a reference drug for the sorbitol assay at concentrations ranging from 0.125 to 32 µM. Growth, sterility, and solvent control wells were also included for each strain tested.

The microplates were incubated at 35 °C for 24 h, 48 h (*Candida* spp., *Cryptococcus neoformans*, *Aspergillus* spp.), or 72 h (*Trichophyton* spp., *Microsporum* spp.).

The MIC was visually determined as the lowest concentration at which a prominent decrease in turbidity (approximately 50%) was observed for yeast, and a slightly hazy decrease in turbidity (approximately 80%) was observed for dermatophytes and Aspergillus, when compared with the drug-free growth control. The lowest concentration inhibiting any discernible growth (MIC_100_) compared with the control well was also evaluated. For amphotericin B, the MIC_100_ was defined.

### 4.4. Cells Viability Assay

A methylene blue staining was performed with some modifications [93,94]. Briefly, *C. albicans* cells (1 × 10^6^ cells/mL) were exposed to different TG concentrations (2–64 µM) in PBS. After 6 h of incubation, the cells were centrifuged for 5 min at 5000× *g*, then washed two times with PBS and resuspended in PBS. Then, 100 μL of the suspension was mixed with 100 μL methylene blue (0.1 mg/mL stock solution, dissolved at 0.1% in PBS) and incubated for 1–3 min at room temperature. Dead cells were evaluated microscopically (Leitz Laborlux ×320, Leitz Italiana SRL, Milan, Italy) by counting at least 200 cells per field in 5 consecutive visual fields. The values were presented as the percentage of dead cells, concerning the whole cell population. Reported values are mean ± SD of three independent measurements in duplicate.

### 4.5. Membrane Permeabilization

#### 4.5.1. Sytox Green Assay

The Sytox Green assay was performed, as previously reported [95], on *C. albicans* ATCC10231. Approximately 5 × 10^6^ cells/mL were incubated with 1 μM Sytox Green in NaPB for 5 min in the dark. After peptide/antibiotic addition, changes in fluorescence intensity (λ_exc_ = 485 nm, λ_ems_ = 535 nm) caused by the binding of the dye to intracellular DNA were monitored for 120 min for TG and 24 h for amphotericin B in a microplate reader (Infinite M200, Tecan, Salzburg, Austria) at 30 °C. Controls were cells not treated with the peptides.

#### 4.5.2. PI Uptake Assay

Membrane permeability of *Candida* cells after TG treatment was evaluated using a PI uptake assay with some modifications [96,97]. In brief, an overnight culture of *C. albicans* ATCC10231 in Sabouraud Dextrose Broth was harvested by centrifugation and washed two times with 10 mM PBS solution. TG (16-32-64 µM concentrations) was added at 1 mL of cell suspension (1 *×* 10^7^ cells/mL) and then incubated for 6 h at 30 °C. The cells were washed in PBS, resuspended in PBS and incubated at 28 °C with 5 μg/mL PI (1 mg/mL stock solution) (Merk Life Science S.r.l. Milan, Italy) in the dark for 15 min. Then, 50 µL of cells were spotted on a microscope slide, covered with a coverslip, and PI fluorescence was examined from at least 200 cells in one sample under a fluorescence microscope (Leitz Aristoplan ×400, Leitz Italiana Srl, Milan, Italy) using a Texas red filter, λ_ex_ = 490 nm, λ_em_ = 630 nm. The percentage (%) of PI uptake was evaluated as the percent of red fluorescent stained cells in the sample with respect to the untreated control cells. Isopropyl alcohol (50%) was used as a positive control and induced 97 ± 1.72 % of dead cells. Three replicates were performed and values were reported as mean ± SD.

### 4.6. Ergosterol Binding Assay

TG MIC values on *C. albicans* ATCC10231 were evaluated according to the standard broth microdilution method (CLSI) in the absence and presence of exogenous ergosterol (Merk Life Science S.r.l., Milan, Italy), at different concentration (50–150 µg/mL) added to the RPMI 1640 medium. The plates were incubated at 37 °C, and the MIC_100_ was determined after 48 h of incubation. Amphotericin B was used as the positive control [43].

### 4.7. Sorbitol Effect Assay

The MIC of TG against *C. albicans* ATCC10231 was determined in RPMI 1640 medium according to the CLSI broth microdilution method [33], with and without 0.8 M sorbitol (Sigma Aldrich, St. Louis, MO, USA) used as a fungal cell wall osmoprotectant [41,98]. The plates were incubated at 35 °C and absorbance read after 48 h. MICs were defined as the lowest concentrations capable of visually inhibiting 100% of fungal growth. Experiments were performed in triplicate, and caspofungin was used as the positive control.

### 4.8. Effect of TG on C. albicans Virulence Factors

#### 4.8.1. Yeast–Mycelial Conversion Assay

We evaluated the first step of yeast–mycelial conversion by studying germ tube formation. The test was performed at 37 °C in a medium that promotes germ tube formation containing N-acetyl-D-glucosamine (GlcNAc), a morphogenic effector in *C. albicans* and other nutritional inducers [99]. The medium was prepared as follows: N-acetylglucosamine 500 mg, L-alanine 50 mg, L-biotin 0.05 mg, L-proline 50 mg (all reagents were purchased by Merck Life Science S.r.l., Milan, Italy), 0.2 M imidazole-HCl buffer (pH 6.6) 25 mL, distilled water up to 500 mL. The dimorphic switching, evaluated as germ tubes formation, was evaluated using a protocol described elsewhere, with minor modifications [100,101]. Briefly, a culture of *C. albicans* ATCC10231 grown overnight at 30 °C in Yeast Peptone Dextrose Broth (YPDB) was harvested by centrifugation (5000× *g*/5 min), washed twice in PBS, and resuspended to 1 *×* 10^6^–5 *×* 10^6^ cells/mL in GlcNAc solution. Then, 100 µL of the working suspension was added to 900 µL of GlcNAc containing TG concentrations ranging from 0.125 to 64 µM. Control without substance was also included. After 3 h of incubation at 37 °C under shaking, the percentage of germ tubes with respect to the total cells was evaluated by counting about 250 cells from each sample with an optical microscope (Leitz Laborlux ×320, Leitz Italiana Srl, Milan, Italy). In the control without substance, germ tubes were 80–90%. Three independent experiments were performed in duplicate.

#### 4.8.2. Anti-Biofilm Assay

The effect of TG on *C. albicans* biofilm was evaluated at two different phases (formation and mature biofilm) according to the protocol of Pierce et al. [102], with some modifications. Briefly, *C. albicans* cells obtained from an overnight culture in YPDB at 30 °C with shaking were washed twice in cold PBS and resuspended in a prewarmed RPMI 1640 medium, at a final cell density of 10^6^ cells/mL (inoculum suspension). The procedures were divided into two steps: a biofilm formation step, and mature biofilm step. For inhibition of biofilm formation, 200 µL of inoculum suspension was added in duplicate to a 96-well flat bottom microtiter plate and incubated for 90 min at 37 °C; then the medium was aspirated, and the wells washed gently with PBS. Then, 200 µL of TG at different concentrations (from 4 to 64 µM) in RPMI 1640 medium was added to the wells. The plates were incubated at 37 °C for 24 h [103].

As a negative control, some wells contained only RPMI medium; and as the positive control, some wells contained cells without TG. The plates were then incubated at 37 °C for 24 h without shaking.

In mature biofilm, 200 µL of inoculum suspension was transferred into each well of microtiter plates and then incubated at 37 °C for 24 h. Afterwards, the medium was gently aspirated, the wells were washed twice with PBS to remove non-adherent cells and 200 µL of TG was added. The microplate was incubated for 24 h at 37 °C. At 24 h of incubation, the medium was aspirated and biofilms were washed twice with PBS.

Two methods were performed to evaluate biofilm production: XTT reduction assay, and CV staining.

The XTT reduction assay [102] was performed to evaluate the activity of TG on the biofilm (formation step and mature step), by measuring the metabolic activity of cells within the biofilm. Tetrazolium salt 2,3-bis-[2-methyloxy-4-nitro-5-sulfophenyl]-2H-tetrazolium-5-carboxanilide (XTT, Sigma-Aldrich Corp., St. Louis, MO, USA) was dissolved in PBS at a concentration of 0.5 g/L. The solution was filter-sterilized and stored at −70 °C until use. Menadione solution (10 mM stock solution in acetone; Sigma-Aldrich Corp., St. Louis, MO, USA) was prepared immediately before each experiment. For each assay, XTT solution was thawed and mixed with menadione solution (final concentration 1 µM). Then, 100 µL of XTT-menadione solution was transferred to each prewashed biofilm and to control wells of 96-well plates. The plates were covered with aluminum foil and incubated in the dark at 37 °C for 2 h. Then, 80 µL of the solution was transferred to each well of new 96-well plates, and colorimetric change was evaluated at 490 nm using a microtiter plate reader (Thermo Electron Type 355 MultiskanEX, Vantaa, Finland). Three experiments in duplicate were performed and the data were expressed as the percentage of reduction of metabolic activity compared to the drug-free control.

Crystal violet staining [104] was performed, with minor modification, to evaluate the effect of TG on biofilm by quantifying biofilm biomass. Briefly, 150 μL of CV (0.5% *w*:*v*) was added to all wells for 30 min. The wells were washed and air dried. Finally, 150 μL of 95% ethanol (Sigma-Aldrich corp., St. Louis, MO, USA) was added for 20 min, to solubilize the bound CV. Destaining solution (100 μL) from each well was transferred to a new microplate, and the absorbance was read at 590 nm using a microplate reader (Thermo Electron Type 355 MultiskanEX, Vantaa, Finland). Two experiments in triplicate were performed, and the data were expressed as the percentage of biofilm biomass inhibition compared to drug-free control.

### 4.9. Statistical Analysis

Data obtained were analyzed using the mode, range, and mean ± standard deviation of three or four independent experiments in duplicate. An unpaired Student’s *t*-test was used for the statistical analysis, and a value of * *p* < 0.05 was considered statistically significant ** *p* < 0.01 as highly significant.

## Figures and Tables

**Table 1:** MIC values of TG against *Candida* species and *Cryptococcus neoformans*.

	Temporin G								Amphotericin B	
	MIC_50_				MIC_100_				MIC_100_	
	24 h		48 h		24 h		48 h		48 h	
	Mode	Range	Mode	Range	Mode	Range	Mode	Range	Mode	Range
*Candida albicans* ATCC10231	4	4–8	8	4–8	16	8–32	16	8–32	0.25	0.125–0.25
*Candida albicans* ATCC24433	16	16–32	32	32–64	32	32–64	32	32–64	0.25	0.125–0.25
*Candida tropicalis* DSM11953	4	4–8	8	8–16	16	8–16	16	16–32	1	0.5–1
*Candida glabrata* PMC0805	16	16–32	32	32–64	64	64–128	128	128	4	4–8
*Candida parapsilosis* PMC0711	64	32–64	128	128	128	64–128	128	128	1	0.5–2
*Candida parapsilosis* DSM11224	64	32–64	128	128	128	64–128	128	128	1	0.5–1
*Candida krusei* PMC0610	64	64	128	128	128	64–128	128	128	1	0.5–1
*Candida krusei* PMC0624	16	8–16	32	32–64	32	32–64	32	32–64	1	1–2
*Cryptococcus neoformans* DSM11759	16	16–32	16	16–32	32	16–32	32	16–64	0.5	0.25–0.5
*Candida* spp. Mean ± SD	30 ± 24.5		65.9 ± 48.9		65.5 ± 45.9		80.2 ± 49.6		1.3 ± 1.7	
*Cryptococcus* spp. Mean ± SD	20 ± 7.4		22 ± 8.3		28 ± 7.4		32 ± 14.8		0.4 ± 0.1	

MIC_50_ and MIC_100_ represent the lowest peptide concentration that caused ≥50% and 100% reduction of visual growth, respectively. Four experiments were performed in duplicate, and the modal MIC and Range values were reported. The values are reported in µM.

**Table 2:** MIC values of TG against a panel of filamentous pathogenic fungi.

	Temporin G		Amphotericin B
	MIC80	MIC100	MIC100
*Microsporum gypseum* DSM3824	32	64	0.25
*Microsporum gypseum* PMC7330	32	128	0.25
*Microsporum canis* DSM10708	16	64	0.125
*Trichophyton mentagrophytes* DSM4870	4	16	0.125
*Trichophyton mentagrophytes* PMC6530	32	64	0.25
*Trichophyton mentagrophytes* PMC6529	8	32	0.25
*Trichophyton mentagrophytes* PMC6528	16	32	0.5
*Aspergillus niger* PMC7101	128	128	16
*Aspergillus brasiliensis* DSM1988	128	128	2
*Aspergillus terreus* PMC7201	128	128	16
*Microsporum* spp. Mean ± SD	27.7 ± 13.9	101.3 ± 32.2	0.6 ± 0.6
*Trichophyton* spp. Mean ± SD	19 ± 20.3	42 ± 28.7	0.3 ± 0.1
*Aspergillus* spp. Mean ± SD	112 ± 28.3	124.9 ± 49.6	12.7 ± 9.1

MIC_80_ and MIC_100_ represent the lowest peptide concentration that caused ≥80% and 100% reduction of visual growth, respectively. Results are the modal MIC values of four experiments performed in duplicate. For all MIC values, arithmetic mean ± SD was evaluated. The values are reported in µM.

**Table 3:** MIC values of TG against *C. albicans* ATCC10231 in the presence and absence of sorbitol.

	−SORBITOL	+SORBITOL
Temporin G	21.3 ± 9.2	21.3 ± 9.2
Caspofungin	0.21 ± 0.07	26.7 ± 9.23

Sorbitol at 0.8 M was added to the medium. After 48 h of incubation at 37 °C, the MIC was determined and compared to that found in the absence of sorbitol. Caspofungin was used as control drug. The values are expressed as a mean of three separate experiments ± SD. Data are reported in µM.

## Data Availability

Data sharing is not applicable.

## References

[1] Bongomin F., Gago S., Oladele R.O., Denning D.W. (2017). Global and Multi-National Prevalence of Fungal Diseases-Estimate Precision. J. Fungi.

[2] Kim J.Y. (2016). Human fungal pathogens: Why should we learn?. J. Microbiol..

[3] Denning D.W., Kneale M., Sobel J.D., Rautemaa-Richardson R. (2018). Global burden of recurrent vulvovaginal candidiasis: A systematic review. Lancet. Infect. Dis..

[4] Firacative C. (2020). Invasive fungal disease in humans: Are we aware of the real impact?. Mem. Inst. Oswaldo Cruz.

[5] Schmiedel Y., Zimmerli S. (2016). Common invasive fungal diseases: An overview of invasive candidiasis, aspergillosis, cryptococcosis, and Pneumocystis pneumonia. Swiss Med. Wkly..

[6] Fisher M.C., Hawkins N.J., Sanglard D., Gurr S.J. (2018). Worldwide emergence of resistance to antifungal drugs challenges human health and food security. Science.

[7] Silva S., Rodrigues C.F., Araujo D., Rodrigues M.E., Henriques M. (2017). Candida Species Biofilms’ Antifungal Resistance. J. Fungi.

[8] Kuhn D.M., Ghannoum M.A. (2004). Candida biofilms: Antifungal resistance and emerging therapeutic options. Curr. Opin. Investig. Drugs.

[9] Boman H.G. (2000). Innate immunity and the normal microflora. Immunol. Rev..

[10] Bahar A.A., Ren D. (2013). Antimicrobial peptides. Pharmaceuticals.

[11] Zasloff M. (2002). Antimicrobial peptides of multicellular organisms. Nature.

[12] Bulet P., Stocklin R., Menin L. (2004). Anti-microbial peptides: From invertebrates to vertebrates. Immunol. Rev..

[13] Vanzolini T., Bruschi M., Rinaldi A.C., Magnani M., Fraternale A. (2022). Multitalented Synthetic Antimicrobial Peptides and Their Antibacterial, Antifungal and Antiviral Mechanisms. Int. J. Mol. Sci..

[14] Chianese A., Zannella C., Monti A., De Filippis A., Doti N., Franci G., Galdiero M. (2022). The Broad-Spectrum Antiviral Potential of the Amphibian Peptide AR-23. Int. J. Mol. Sci..

[15] Sato H., Feix J.B. (2006). Peptide-membrane interactions and mechanisms of membrane destruction by amphipathic alpha-helical antimicrobial peptides. Biochim. Biophys. Acta.

[16] Romero S.M., Cardillo A.B., Martinez Ceron M.C., Camperi S.A., Giudicessi S.L. (2020). Temporins: An Approach of Potential Pharmaceutic Candidates. Surg. Infect..

[17] Rinaldi A.C., Mangoni M.L., Rufo A., Luzi C., Barra D., Zhao H., Kinnunen P.K., Bozzi A., Di Giulio A., Simmaco M. (2002). Temporin L: Antimicrobial, haemolytic and cytotoxic activities, and effects on membrane permeabilization in lipid vesicles. Biochem. J..

[18] Raja Z., Andre S., Abbassi F., Humblot V., Lequin O., Bouceba T., Correia I., Casale S., Foulon T., Sereno D. (2017). Insight into the mechanism of action of temporin-SHa, a new broad-spectrum antiparasitic and antibacterial agent. PLoS ONE.

[19] Wade D., Silberring J., Soliymani R., Heikkinen S., Kilpelainen I., Lankinen H., Kuusela P. (2000). Antibacterial activities of temporin A analogs. FEBS Lett..

[20] Marcocci M.E., Amatore D., Villa S., Casciaro B., Aimola P., Franci G., Grieco P., Galdiero M., Palamara A.T., Mangoni M.L. (2018). The Amphibian Antimicrobial Peptide Temporin B Inhibits In Vitro Herpes Simplex Virus 1 Infection. Antimicrob. Agents Chemother..

[21] Simmaco M., Mignogna G., Canofeni S., Miele R., Mangoni M.L., Barra D. (1996). Temporins, antimicrobial peptides from the European red frog Rana temporaria. Eur. J. Biochem..

[22] Rinaldi A.C., Conlon J.M., Kastin A.J. (2013). Chapter 56–Temporins. Handbook of Biologically Active Peptides.

[23] Capparelli R., Romanelli A., Iannaccone M., Nocerino N., Ripa R., Pensato S., Pedone C., Iannelli D. (2009). Synergistic antibacterial and anti-inflammatory activity of temporin A and modified temporin B in vivo. PLoS ONE.

[24] Roscetto E., Bellavita R., Paolillo R., Merlino F., Molfetta N., Grieco P., Buommino E., Catania M.R. (2021). Antimicrobial Activity of a Lipidated Temporin L Analogue against Carbapenemase-Producing *Klebsiella pneumoniae* Clinical Isolates. Antibiotics.

[25] Mangoni M.L., Grazia A.D., Cappiello F., Casciaro B., Luca V. (2016). Naturally Occurring Peptides from Rana temporaria: Antimicrobial Properties and More. Curr. Top. Med. Chem..

[26] De Angelis M., Casciaro B., Genovese A., Brancaccio D., Marcocci M.E., Novellino E., Carotenuto A., Palamara A.T., Mangoni M.L., Nencioni L. (2021). Temporin G, an amphibian antimicrobial peptide against influenza and parainfluenza respiratory viruses: Insights into biological activity and mechanism of action. FASEB J..

[27] Casciaro B., Loffredo M.R., Cappiello F., Fabiano G., Torrini L., Mangoni M.L. (2020). The Antimicrobial Peptide Temporin G: Anti-Biofilm, Anti-Persister Activities, and Potentiator Effect of Tobramycin Efficacy Against Staphylococcus aureus. Int. J. Mol. Sci..

[28] Tyc K.M., Kuhn C., Wilson D., Klipp E. (2014). Assessing the advantage of morphological changes in *Candida albicans*: A game theoretical study. Front. Microbiol..

[29] Tsui C., Kong E.F., Jabra-Rizk M.A. (2016). Pathogenesis of *Candida albicans* biofilm. Pathog. Dis..

[30] Pereira R., Dos Santos Fontenelle R.O., de Brito E.H.S., de Morais S.M. (2021). Biofilm of *Candida albicans*: Formation, regulation and resistance. J. Appl. Microbiol..

[31] Rajendran R., Sherry L., Nile C.J., Sherriff A., Johnson E.M., Hanson M.F., Williams C., Munro C.A., Jones B.J., Ramage G. (2016). Biofilm formation is a risk factor for mortality in patients with *Candida albicans* bloodstream infection-Scotland, 2012–2013. Clin. Microbiol. Infect..

[32] CLSI (2017). Reference Method for Broth Dilution Antifungal Susceptibility Testing of Filamentous Fungi.

[33] CLSI (2017). Reference Method for Broth Dilution Antifungal Susceptibility Testing of Yeasts.

[34] Luca V., Olivi M., Di Grazia A., Palleschi C., Uccelletti D., Mangoni M.L. (2014). Anti-Candida activity of 1–18 fragment of the frog skin peptide esculentin-1b: In vitro and in vivo studies in a Caenorhabditis elegans infection model. Cell. Mol. Life Sci. CMLS.

[35] Casciaro B., Loffredo M.R., Cappiello F., Verrusio W., Corleto V.D., Mangoni M.L. (2020). Frog Skin-Derived Peptides Against Corynebacterium jeikeium: Correlation between Antibacterial and Cytotoxic Activities. Antibiotics.

[36] Odds F.C., Brown A.J., Gow N.A. (2003). Antifungal agents: Mechanisms of action. Trends Microbiol..

[37] Escalante A., Gattuso M., Perez P., Zacchino S. (2008). Evidence for the mechanism of action of the antifungal phytolaccoside B isolated from *Phytolacca tetramera* Hauman. J. Nat. Prod..

[38] Biernasiuk A., Berecka-Rycerz A., Gumieniczek A., Malm M., Laczkowski K.Z., Szymanska J., Malm A. (2021). The newly synthesized thiazole derivatives as potential antifungal compounds against *Candida albicans*. Appl. Microbiol. Biotechnol..

[39] Sentandreu R., Herrero E., Elorza M.V., Rico H., Pastor J. (1983). Synthesis and assembly of wall polymers on regenerating yeast protoplasts. Exp. Suppl..

[40] de Oliveira Filho G.B., Cardoso M.V.O., Espindola J.W.P., Oliveira E.S.D.A., Ferreira R.S., Coelho P.L., Anjos P.S.D., Santos E.S., Meira C.S., Moreira D.R.M. (2017). Structural design, synthesis and pharmacological evaluation of thiazoles against *Trypanosoma cruzi*. Eur. J. Med. Chem..

[41] Chen H., Zhou X., Ren B., Cheng L. (2020). The regulation of hyphae growth in *Candida albicans*. Virulence.

[42] Mukaremera L., Lee K.K., Mora-Montes H.M., Gow N.A.R. (2017). *Candida albicans* Yeast, Pseudohyphal, and Hyphal Morphogenesis Differentially Affects Immune Recognition. Front. Immunol..

[43] Jacobsen I.D., Wilson D., Wachtler B., Brunke S., Naglik J.R., Hube B. (2012). *Candida albicans* dimorphism as a therapeutic target. Expert Rev. Anti-Infect. Ther..

[44] Lo H.J., Kohler J.R., DiDomenico B., Loebenberg D., Cacciapuoti A., Fink G.R. (1997). Nonfilamentous *C. albicans* mutants are avirulent. Cell.

[45] Richardson M.D.W. (2012). Fungal Infection: Diagnosis and Management.

[46] Hallen-Adams H.E., Suhr M.J. (2017). Fungi in the healthy human gastrointestinal tract. Virulence.

[47] Colombo A.L., Guimaraes T., Sukienik T., Pasqualotto A.C., Andreotti R., Queiroz-Telles F., Nouer S.A., Nucci M. (2014). Prognostic factors and historical trends in the epidemiology of candidemia in critically ill patients: An analysis of five multicenter studies sequentially conducted over a 9-year period. Intensive Care Med..

[48] Moran G., Sullivan D., Calderone R.A. (2019). An introduction to the medically important Candida species. Candida and Candidiasis.

[49] Arastehfar A., Carvalho A., Nguyen M.H., Hedayati M.T., Netea M.G., Perlin D.S., Hoenigl M. (2020). COVID-19-Associated Candidiasis (CAC): An Underestimated Complication in the Absence of Immunological Predispositions?. J. Fungi.

[50] Rolling T., Hohl T.M., Zhai B. (2020). Minority report: The intestinal mycobiota in systemic infections. Curr. Opin. Microbiol..

[51] Wadhwa R., Pandey P., Gupta G., Aggarwal T., Kumar N., Mehta M., Satija S., Gulati M., Madan J., Dureja H. (2019). Emerging Complexity and the Need for Advanced Drug Delivery in Targeting Candida Species. Curr. Top. Med. Chem..

[52] Wall G., Montelongo-Jauregui D., Vidal Bonifacio B., Lopez-Ribot J.L., Uppuluri P. (2019). *Candida albicans* biofilm growth and dispersal: Contributions to pathogenesis. Curr. Opin. Microbiol..

[53] Gulati M., Nobile C.J. (2016). *Candida albicans* biofilms: Development, regulation, and molecular mechanisms. Microbes Infect..

[54] Kernien J.F., Snarr B.D., Sheppard D.C., Nett J.E. (2017). The Interface between Fungal Biofilms and Innate Immunity. Front. Immunol..

[55] Cavalheiro M., Teixeira M.C. (2018). Candida Biofilms: Threats, Challenges, and Promising Strategies. Front. Med..

[56] Chandra J., Mukherjee P.K., Ghannoum M.A. (2012). Candida biofilms associated with CVC and medical devices. Mycoses.

[57] Tumbarello M., Fiori B., Trecarichi E.M., Posteraro P., Losito A.R., De Luca A., Sanguinetti M., Fadda G., Cauda R., Posteraro B. (2012). Risk factors and outcomes of candidemia caused by biofilm-forming isolates in a tertiary care hospital. PLoS ONE.

[58] Vitalis E., Nagy F., Toth Z., Forgacs L., Bozo A., Kardos G., Majoros L., Kovacs R. (2020). Candida biofilm production is associated with higher mortality in patients with candidaemia. Mycoses.

[59] Stewart P.S., Costerton J.W. (2001). Antibiotic resistance of bacteria in biofilms. Lancet.

[60] Kowalski C.H., Morelli K.A., Schultz D., Nadell C.D., Cramer R.A. (2020). Fungal biofilm architecture produces hypoxic microenvironments that drive antifungal resistance. Proc. Natl. Acad. Sci. USA.

[61] Costa-de-Oliveira S., Rodrigues A.G. (2020). *Candida albicans* Antifungal Resistance and Tolerance in Bloodstream Infections: The Triad Yeast-Host-Antifungal. Microorganisms.

[62] Garcia-Rubio R., Cuenca-Estrella M., Mellado E. (2017). Triazole Resistance in Aspergillus Species: An Emerging Problem. Drugs.

[63] Alexander B.D., Johnson M.D., Pfeiffer C.D., Jimenez-Ortigosa C., Catania J., Booker R., Castanheira M., Messer S.A., Perlin D.S., Pfaller M.A. (2013). Increasing Echinocandin Resistance in *Candida glabrata*: Clinical Failure Correlates with Presence of FKS Mutations and Elevated Minimum Inhibitory Concentrations. Clin. Infect. Dis..

[64] Peyclit L., Yousfi H., Rolain J.M., Bittar F. (2021). Drug Repurposing in Medical Mycology: Identification of Compounds as Potential Antifungals to Overcome the Emergence of Multidrug-Resistant Fungi. Pharm. Base.

[65] Taylor G. (2015). The importance of stewardship. Can. Commun. Dis. Rep..

[66] World Health Organization (2014). Antimicrobial Resistance Global Report on Surveillance: 2014 Summary.

[67] Geddes-McAlister J., Shapiro R.S. (2019). New pathogens, new tricks: Emerging, drug-resistant fungal pathogens and future prospects for antifungal therapeutics. Annu. N. Y. Acad. Sci..

[68] Mangoni M.L., Miele R., Renda T.G., Barra D., Simmaco M. (2001). The synthesis of antimicrobial peptides in the skin of Rana esculenta is stimulated by microorganisms. FASEB J..

[69] Grieco P., Carotenuto A., Auriemma L., Saviello M.R., Campiglia P., Gomez-Monterrey I.M., Marcellini L., Luca V., Barra D., Novellino E. (2013). The effect of D-amino acid substitution on the selectivity of temporin L towards target cells: Identification of a potent anti-*Candida* peptide. Biochim. Biophys. Acta (BBA)-Biomembr..

[70] Bellavita R., Maione A., Merlino F., Siciliano A., Dardano P., De Stefano L., Galdiero S., Galdiero E., Grieco P., Falanga A. (2022). Antifungal and Antibiofilm Activity of Cyclic Temporin L Peptide Analogues against Albicans and Non-Albicans Candida Species. Pharmaceutics.

[71] Brunet K., Verdon J., Ladram A., Arnault S., Rodier M.H., Cateau E. (2022). Antifungal activity of [K^3^] temporin-SHa against medically relevant yeasts and moulds. Can. J. Microbiol..

[72] Iyer K.R., Revie N.M., Fu C., Robbins N., Cowen L.E. (2021). Treatment strategies for cryptococcal infection: Challenges, advances and future outlook. Nat. Rev. Microbiol..

[73] Sahoo A.K., Mahajan R. (2016). Management of tinea corporis, tinea cruris, and tinea pedis: A comprehensive review. Indian Dermatol. Online J..

[74] Khurana A., Sardana K., Chowdhary A. (2019). Antifungal resistance in dermatophytes: Recent trends and therapeutic implications. Fungal Genet. Biol..

[75] Reichert-Lima F., Lyra L., Pontes L., Moretti M.L., Pham C.D., Lockhart S.R., Schreiber A.Z. (2018). Surveillance for azoles resistance in Aspergillus spp. highlights a high number of amphotericin B-resistant isolates. Mycoses.

[76] Lima P.G., Souza P.F.N., Freitas C.D.T., Bezerra L.P., Neto N.A.S., Silva A.F.B., Oliveira J.T.A., Sousa D.O.B. (2021). Synthetic peptides against *Trichophyton mentagrophytes* and *T. rubrum*: Mechanisms of action and efficiency compared to griseofulvin and itraconazole. Life Sci..

[77] Cavalcante C.S., Falcao C.B., Fontenelle R.O., Andreu D., Radis-Baptista G. (2017). Anti-fungal activity of Ctn [15–34], the C-terminal peptide fragment of crotalicidin, a rattlesnake venom gland cathelicidin. J. Antibiot..

[78] Zheng Y.J., Xie T., Wu L., Liu X.Y., Zhu L., Chen Y., Mao E.Q., Han L.Z., Chen E.Z., Yang Z.T. (2021). Epidemiology, species distribution, and outcome of nosocomial Candida spp. bloodstream infection in Shanghai: An 11-year retrospective analysis in a tertiary care hospital. Annu. Clin. Microbiol. Antimicrob..

[79] Mahalka A.K., Kinnunen P.K. (2009). Binding of amphipathic alpha-helical antimicrobial peptides to lipid membranes: Lessons from temporins B and L. Biochim. Biophys. Acta.

[80] Biswas S., Van Dijck P., Datta A. (2007). Environmental sensing and signal transduction pathways regulating morphopathogenic determinants of *Candida albicans*. Microbiol. Mol. Biol. Rev..

[81] Mitchell A.P. (1998). Dimorphism and virulence in *Candida albicans*. Curr. Opin. Microbiol..

[82] Saville S.P., Lazzell A.L., Monteagudo C., Lopez-Ribot J.L. (2003). Engineered control of cell morphology in vivo reveals distinct roles for yeast and filamentous forms of *Candida albicans* during infection. Eukaryot. Cell..

[83] Carlisle P.L., Banerjee M., Lazzell A., Monteagudo C., Lopez-Ribot J.L., Kadosh D. (2009). Expression levels of a filament-specific transcriptional regulator are sufficient to determine *Candida albicans* morphology and virulence. Proc. Natl. Acad. Sci. USA.

[84] Wachtler B., Wilson D., Haedicke K., Dalle F., Hube B. (2011). From Attachment to Damage: Defined Genes of *Candida albicans* Mediate Adhesion, Invasion and Damage during Interaction with Oral Epithelial Cells. PLoS ONE.

[85] Lorenz M.C., Bender J.A., Fink G.R. (2004). Transcriptional response of *Candida albicans* upon internalization by macrophages. Eukaryot. Cell.

[86] Richard M.L., Nobile C.J., Bruno V.M., Mitchell A.P. (2005). *Candida albicans* biofilm-defective mutants. Eukaryot. Cell.

[87] Ramage G., VandeWalle K., Lopez-Ribot J.L., Wickes B.L. (2002). The filamentation pathway controlled by the Efg1 regulator protein is required for normal biofilm formation and development in *Candida albicans*. Fems. Microbiol. Lett..

[88] Chandra J., Kuhn D.M., Mukherjee P.K., Hoyer L.L., McCormick T., Ghannoum M.A. (2001). Biofilm formation by the fungal pathogen *Candida albicans*: Development, architecture, and drug resistance. J. Bacteriol..

[89] Deveau A., Hogan D.A. (2011). Linking Quorum Sensing Regulation and Biofilm Formation by *Candida albicans*. Quor. Sens. Methods Protoc..

[90] Saville S.P., Lazzell A.L., Bryant A.P., Fretzen A., Monreal A., Solberg E.O., Monteagudo C., Lopez-Ribot J.L., Milne G.T. (2006). Inhibition of filamentation can be used to treat disseminated candidiasis. Antimicrob. Agents Chemother..

[91] Vila T., Romo J.A., Pierce C.G., McHardy S.F., Saville S.P., Lopez-Ribot J.L. (2017). Targeting *Candida albicans* filamentation for antifungal drug development. Virulence.

[92] Souza P.F.N., Marques L.S.M., Oliveira J.T.A., Lima P.G., Dias L.P., Neto N.A.S., Lopes F.E.S., Sousa J.S., Silva A.F.B., Caneiro R.F. (2020). Synthetic antimicrobial peptides: From choice of the best sequences to action mechanisms. Biochimie.

[93] Painting K., Kirsop B. (1990). A Quick Method for Estimating the Percentage of Viable Cells in a Yeast Population, Using Methylene-Blue Staining. World J. Microb. Biot..

[94] Van Zandyke S.M., Simal O., Gualdoni S., Smart K.A., Smart K.A. (2003). Yeast quality and fluorophore technologies. Brewing Yeast Fermentation Performance.

[95] Merlino F., Carotenuto A., Casciaro B., Martora F., Loffredo M.R., Di Grazia A., Yousif A.M., Brancaccio D., Palomba L., Novellino E. (2017). Glycine-replaced derivatives of [Pro(3),DLeu(9)]TL, a temporin L analogue: Evaluation of antimicrobial, cytotoxic and hemolytic activities. Eur. J. Med. Chem..

[96] D’Auria F.D., Tecca M., Scazzocchio F., Renzini V., Strippoli V. (2003). Effect of propolis on virulence factors of *Candida albicans*. J. Chemother..

[97] Li R.F., Yan X.H., Lu Y.B., Lu Y.L., Zhang H.R., Chen S.H., Liu S., Lu Z.F. (2015). Anti-candidal activity of a novel peptide derived from human chromogranin A and its mechanism of action against *Candida krusei*. Exp. Ther. Med..

[98] Frost D.J., Brandt K.D., Cugier D., Goldman R. (1995). A whole-cell *Candida albicans* assay for the detection of inhibitors towards fungal cell wall synthesis and assembly. J. Antibiot..

[99] Cassone A., Sullivan P.A., Shepherd M.G. (1985). N-acetyl-D-glucosamine-induced morphogenesis in *Candida albicans*. Microbiologica.

[100] Simonetti N., Strippoli V., Cassone A. (1974). Yeast-mycelial conversion induced by N-acetyl-D-glucosamine in *Candida albicans*. Nature.

[101] D’Auria F.D., Laino L., Strippoli V., Tecca M., Salvatore G., Battinelli L., Mazzanti G. (2001). In vitro activity of tea tree oil against *Candida albicans* mycelial conversion and other pathogenic fungi. J. Chemother..

[102] Pierce C.G., Uppuluri P., Tristan A.R., Wormley F.L., Mowat E., Ramage G., Lopez-Ribot J.L. (2008). A simple and reproducible 96-well plate-based method for the formation of fungal biofilms and its application to antifungal susceptibility testing. Nat. Protoc..

[103] Liu R.H., Shang Z.C., Li T.X., Yang M.H., Kong L.Y. (2017). In Vitro Antibiofilm Activity of Eucarobustol E against *Candida albicans*. Antimicrob. Agents Chemother..

[104] Melo A.S., Bizerra F.C., Freymuller E., Arthington-Skaggs B.A., Colombo A.L. (2011). Biofilm production and evaluation of antifungal susceptibility amongst clinical Candida spp. isolates, including strains of the *Candida parapsilosis* complex. Med. Mycol..

